# Supplementary material for: Blood Metabolites Associate with Prognosis in Endometrial Cancer
Source: Metabolites. 2019 Dec 14;9(12):302. doi: 10.3390/metabo9120302 (PMC6949989; doi:10.3390/metabo9120302)
Supplement: Supplementary file 1 [file metabolites-09-00302-s001.pdf]

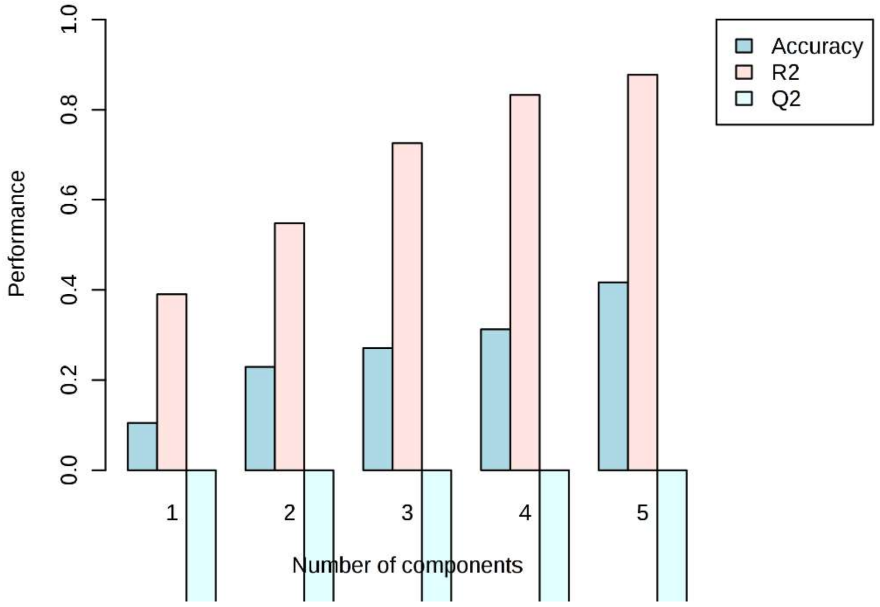

(a)

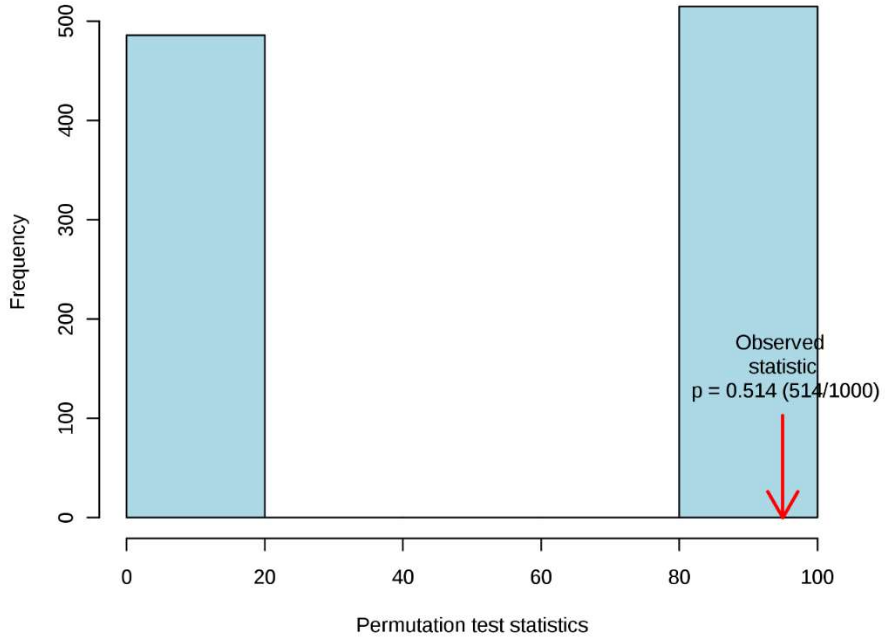

(b)

Figure S1. Cross validation and permutation for the PLS-DA according to survival. (a) The Q2 in the cross validation was -1.19, -1.39, -1.36, -1.27, and -0.98 for 1-5 components, respectively, and **(b)** the permutation test provided a p = 0.51.

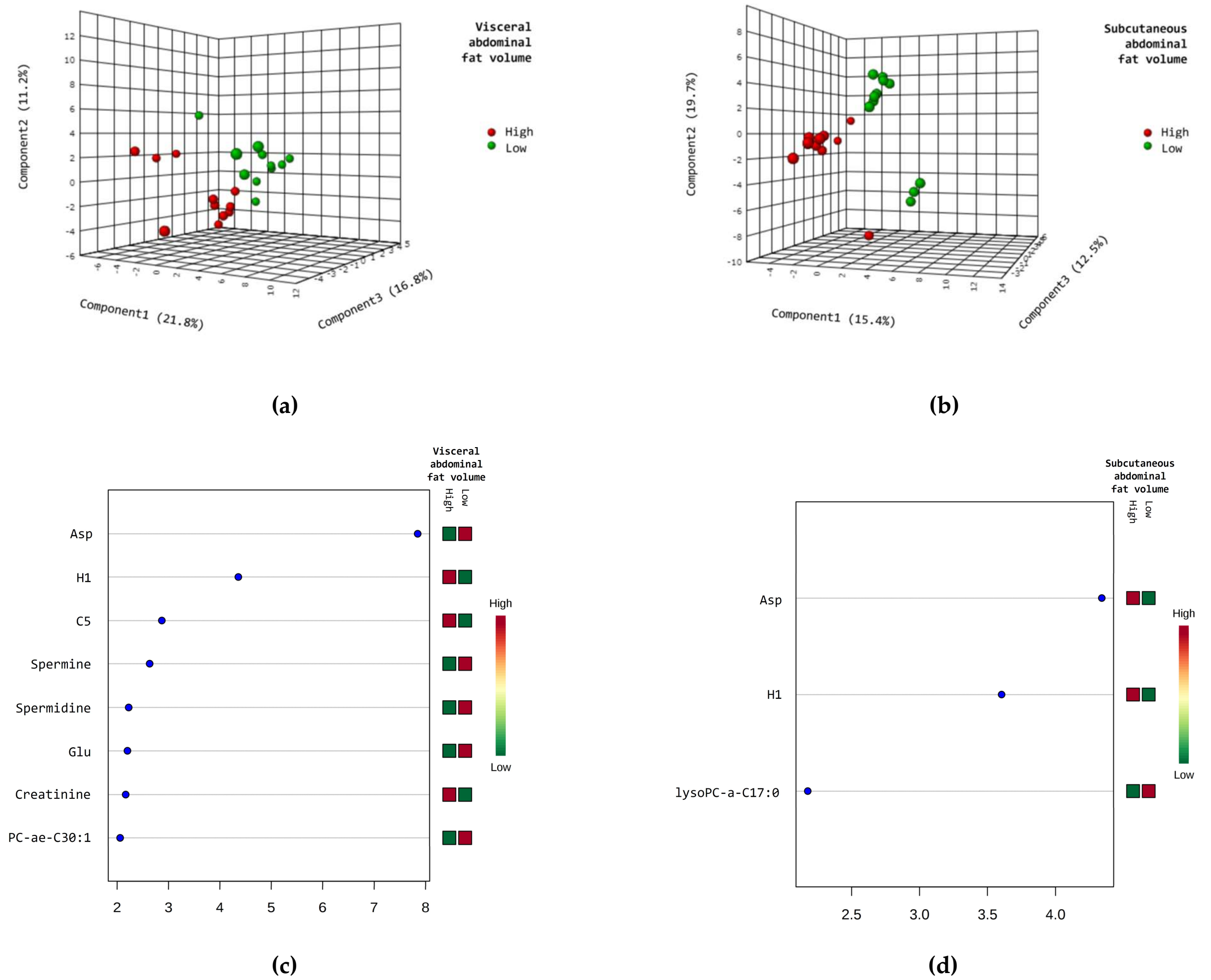

Figure S2. Metabolites in relation with abdominal fat volume in a sub-cohort of patients (n=22). The sub-cohort is separated according to high (red: above median) vs. low (green: below median) visceral abdominal fat volume (cm^3) (**a**) and subcutaneous abdominal fat volume (cm^3) (**b**) through Partial Least Square Discriminant Analyses based on all metabolites; and by visceral abdominal fat volume (cm^3) (**c**) and subcutaneous abdominal fat volume (cm^3) (**d**) demonstrating Variable importance in projection (VIP) metabolites with VIP scores >2.0.

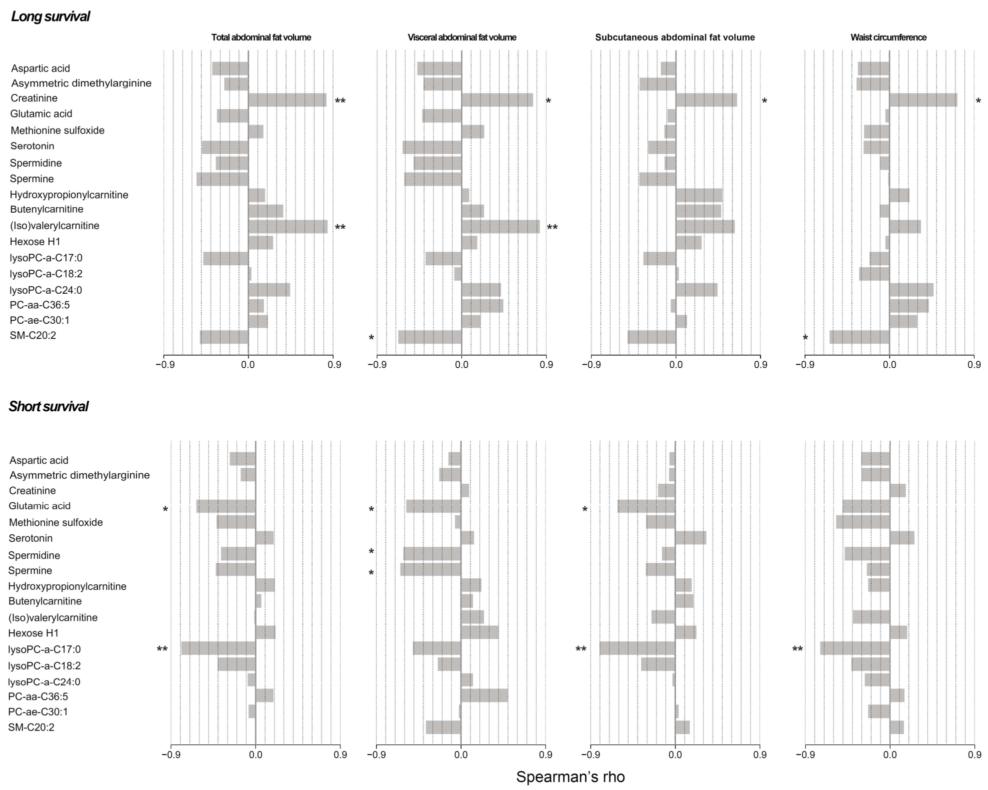

Figure S3. Associations between important metabolites and CT variables. Spearman's rho of ranked values of the selected plasma metabolites with total abdominal fat volume, visceral abdominal fat volume, subcutaneous abdominal fat volume, and waist circumference are illustrated. The upper panel shows correlations in patients with long survival, whereas the lower panel shows correlations in patients with short survival. *P<0.05; **P<0.001.
